# The cytosolic N-terminal domain of V-ATPase a-subunits is a regulatory hub targeted by multiple signals

**DOI:** 10.3389/fmolb.2023.1168680

**Published:** 2023-06-16

**Authors:** Farzana Tuli, Patricia M. Kane

**Affiliations:** Department of Biochemistry and Molecular Biology, SUNY Upstate Medical University, Syracuse, NY, United States

**Keywords:** organelle, acidification, V-ATPase, regulation, a-subunit, isoform, reversible disassembly

## Abstract

Vacuolar H^+^-ATPases (V-ATPases) acidify several organelles in all eukaryotic cells and export protons across the plasma membrane in a subset of cell types. V-ATPases are multisubunit enzymes consisting of a peripheral subcomplex, V_1_, that is exposed to the cytosol and an integral membrane subcomplex, V_o_, that contains the proton pore. The V_o_ a-subunit is the largest membrane subunit and consists of two domains. The N-terminal domain of the a-subunit (aNT) interacts with several V_1_ and V_o_ subunits and serves to bridge the V_1_ and V_o_ subcomplexes, while the C-terminal domain contains eight transmembrane helices, two of which are directly involved in proton transport. Although there can be multiple isoforms of several V-ATPase subunits, the a-subunit is encoded by the largest number of isoforms in most organisms. For example, the human genome encodes four a-subunit isoforms that exhibit a tissue- and organelle-specific distribution. In the yeast S. *cerevisiae*, the two a-subunit isoforms, Golgi-enriched Stv1 and vacuolar Vph1, are the only V-ATPase subunit isoforms. Current structural information indicates that a-subunit isoforms adopt a similar backbone structure but sequence variations allow for specific interactions during trafficking and in response to cellular signals. V-ATPases are subject to several types of environmental regulation that serve to tune their activity to their cellular location and environmental demands. The position of the aNT domain in the complex makes it an ideal target for modulating V_1_-V_o_ interactions and regulating enzyme activity. The yeast a-subunit isoforms have served as a paradigm for dissecting interactions of regulatory inputs with subunit isoforms. Importantly, structures of yeast V-ATPases containing each a-subunit isoform are available. Chimeric a-subunits combining elements of Stv1NT and Vph1NT have provided insights into how regulatory inputs can be integrated to allow V-ATPases to support cell growth under different stress conditions. Although the function and distribution of the four mammalian a-subunit isoforms present additional complexity, it is clear that the aNT domains of these isoforms are also subject to multiple regulatory interactions. Regulatory mechanisms that target mammalian a-subunit isoforms, and specifically the aNT domains, will be described. Altered V-ATPase function is associated with multiple diseases in humans. The possibility of regulating V-ATPase subpopulations via their isoform-specific regulatory interactions are discussed.

## 1 The V_o_ a-subunit in the context of V-ATPase structure and function

Eukaryotic V-ATPases are both remarkably versatile and remarkably conserved. Their versatility is evident in the wide range of functions that they impact directly or indirectly. They are present in virtually all eukaryotic cells where they drive acidification of vacuoles/lysosomes, endosomes, the Golgi apparatus, and regulated secretory vesicles (Forgac, 2007). In these locations, the pH gradient helps to drive secondary transport of ions, amino acids, and other metabolites. V-ATPases create an environment within organelles that supports essential functions; for example, hydrolytic enzymes are activated at the low pH of the lysosome and only operate optimally at this pH. In addition, V-ATPases are recruited to more specialized functions in specific tissues. In neurons, they are essential for neurotransmitter loading into synaptic vesicles; different neurotransmitters rely on either the pH gradient or membrane potential generated by electrogenic proton transport (Farsi et al., 2016). In kidney, V-ATPases are localized at the plasma membrane of the distal renal tubule, where they catalyze export of protons into the urine and thus regulate pH in the blood (Breton and Brown, 2013). In bone, they are recruited to a defined region of the plasma membrane of osteoclasts where they export protons and catalyze bone resorption (Chu et al., 2021). Each of these functions represents variations on a basic mechanism of ATP-driven proton transport, but both the tuning of organelle pH and the recruitment of V-ATPases to the plasma membrane suggest there must be multiple levels of information for localization and regulation present.

**FIGURE 1:**
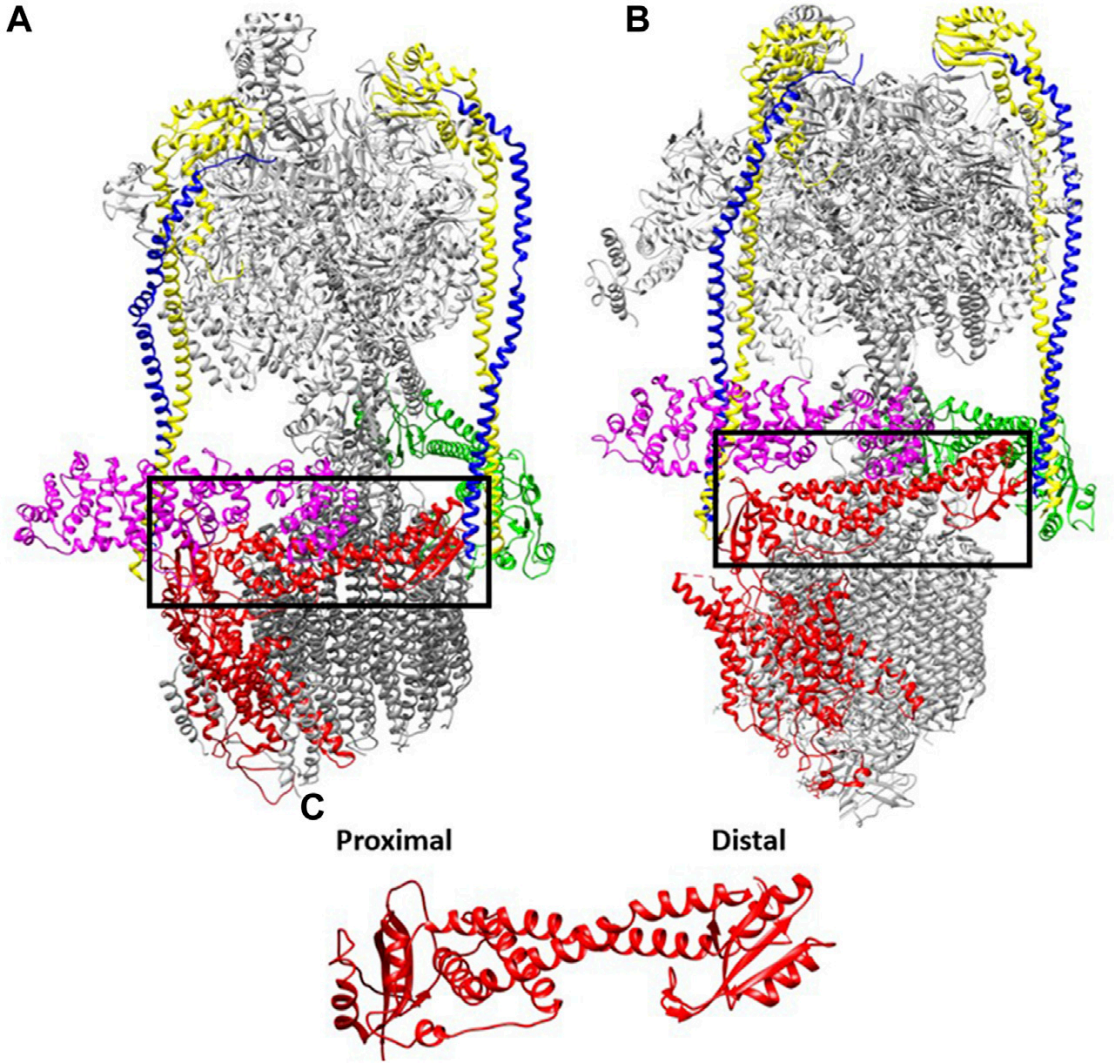
Interactions of the V_1_ subunits with the a-subunit is conserved from yeast to human. The cryoEM maps of V-ATPases from: **(A)** yeast (7FDA, 4.20 Å) and **(B)** human (6WM2, 3.10 Å) e (Khan et al., 2022; L. Wang et al., 2020). The cytosolic NT domain of the a-subunit (marked in black box) harbors many sets of interaction with subunits of V_1_ domain (V1-E and G subunits are marked in yellow and blue respectively, V_1_ H is marked in purple whereas green represents the V_1_ C subunit. **(C)**. The aNT (yeast) domain is zoomed in with the two globular ends defined.

In this context, the conservation of V-ATPase structure seems almost paradoxical. Cryo-EM structures of human and yeast V-ATPases support a very high degree of similarity in subunit composition and organization (Figures 1A,B) (Khan et al., 2022; L. Wang et al., 2020). Some subunit sequences have a high sequence identity across evolutionarily distant organisms, but even those subunits with less sequence conservation can be exchanged between organisms and generate functional V-ATPase complexes in many cases.

All V-ATPases are rotary motors comprised of two subcomplexes, the peripheral V_1_ subcomplex containing the sites of ATP hydrolysis and the integral membrane V_o_ subcomplex that contains the proton pore. Unlike their evolutionary relatives, the A- and F-type ATPases, eukaryotic V-ATPases generally operate as dedicated ATP-driven proton pumps rather than ATP synthases. ATP hydrolysis occurs primarily at sites in the V_1_ A catalytic subunits. Three A-subunits alternate with three B-subunits in the catalytic headgroup of V_1_. In eukaryotic V-ATPases, three peripheral stalks act as stators, ensuring that conformational changes generated by ATP hydrolysis can productively drive rotation of the central rotor and the ring of proteolipid subunits in the V_o_ sector. The peripheral stalks consist of, EG subunit heterodimers that extend along the full length of the catalytic headgroup. At the bottom of the headgroup, the symmetry is broken as the three EG heterodimers make distinct interactions with V_1_ C and H subunits and the N-terminal domain of the V_o_ a-subunit (aNT).

The V_o_ a-subunit is itself a two-domain protein. Its N-terminal half is exposed to the cytosol and acts as part of the stator via multiple interactions with the V_1_ E, G, C, and H-subunits. This aNT domain is visualized in cryo-EM structures as a dumb-bell shape, with two globular ends connected by a coiled-coil (Figure 1C). All a-subunits are likely to adopt this basic shape. The two ends of the aNT domain have been designated “proximal” and “distal”, and each end interacts with one of the peripheral stalks in the assembled V-ATPase. These interactions have been described in detail in yeast (Oot and Wilkens, 2012; Oot et al., 2017; Sharma et al., 2019), and structures of the mammalian V-ATPases contain a similar structural arrangement. The aCT domain consists of eight transmembrane helices, two of which are longer and lie almost horizontally in the membrane (Mazhab-Jafari et al., 2016; Stam and Wilkens, 2017; Roh et al., 2018). The aCT domain contributes to two hemichannels for entry of protons from the cytosol and exit into the organelle lumen, respectively. The separation between the two channels within aCT effectively allows gating of H^+^ transport. Passive transport across the two hemichannels is blocked by interactions between helix 7 and 8 and the c-ring (Roh et al., 2020). Overall, the conservation in structure of a-subunits is not surprising, given its many interactions with other V_1_ and V_o_ subunits and its critical functional role in ATP-driven transport.

However, despite their conserved overall structures, a-subunits of yeast and human show only about 35%–40% overall sequence identity, and even a-subunit isoforms in a single organism usually show only ∼45–60% identity (Nishi and Forgac, 2000; Oka, Toyomura, et al., 2001). Regions of the aCT involved directly in proton transport are more highly conserved, as are certain other regions involved in subunit-subunit interaction. However, there are regions of rather poor similarity that may offer an opportunity for distinct modes of regulation and V-ATPase localization in different cellular locations and between organisms.

## 2 Isoforms of the V_o_ a-subunit

While many organisms encode multiple isoforms of V-ATPase subunits, in most cases, the a-subunit has the largest number of isoforms. *Paramecium* encodes an amazing 17 different a-subunit isoform genes (Wassmer et al., 2006), mammals generally contain 4 a-subunit isoforms (Oka, et al., 2001), A. *thaliana* has 3 (Dettmer et al., 2006), and *S. cerevisiae* has two (Manolson et al., 1994). Consistent with the diverse physiological roles of V-ATPase, the a-subunit isoforms are implicated in directing V-ATPases to distinct intracellular locations, responding to different signals, and supporting tissue-specific functions.

The current model is that all a-subunit isoforms are imported into the ER, folded, and undergo initial steps of assembly with other V_o_, and possibly V_1_, subunits in the ER (Graham et al., 1998; Kane et al., 1999; Malkus et al., 2004). In axons, the V_1_ and V_o_ domains are transported separately at different rates. The assembly of the V_1_ and V_o_ domains occur only when they arrive in torpedo nerve endings (Morel et al., 1998). Dedicated V-ATPase assembly factors that localize to the ER are conserved across yeast, plants, and humans (Graham et al., 1998; Neubert et al., 2008). In yeast, loss of these assembly factors abolishes all V-ATPase activity (Hill and Stevens, 1994; 1995). In humans, mutations in Vma21, one of these assembly factors, compromises V-ATPase activity and causes X-linked myopathy with excess autophagy (Ramachandran et al., 2013; Cannata Serio et al., 2020). After exit from the ER, it is likely that most newly synthesized V-ATPase complexes, either fully or partially assembled, are transported from ER to Golgi. However, some V-ATPases are transported directly from the ER to the vacuole in plants (Viotti et al., 2013). From the Golgi, information in the N-terminal domain of a-subunit isoforms appears to direct distribution of V-ATPases among organelles and the plasma membrane. As described below, targeting of the yeast Stv1 and Vph1 a-subunit isoforms to the Golgi/endosome and vacuole, respectively, is determined by information in the aNT domain (Kawasaki-Nishi et al., 2001a). Recently, a signal necessary and sufficient for Golgi/early endosome localization of the plant a1 isoform was localized to an ∼50 amino acid region in the distal domain of the a1NT (Lupanga et al., 2020). However, the aNT sequences dictating V-ATPase trafficking are known in only a few cases (Finnigan et al., 2011).

Given the diversity of a-subunit isoforms in many organisms, it is worth noting that some organisms survive and thrive with only one a-subunit isoform. For example, although S. *cerevisiae* and a number of other fungi have two a-subunit isoforms, the fission yeast S. *pombe* and filamentous fungus N. *crassa* have only one (Chavez et al., 2006). Even among multicellular plants, Lupanga et al. (2020) showed that the liverwort, *Marchantia*, has only one a-subunit isoform that localizes to the Golgi, early endosome and vacuole (Lupanga et al., 2020). Finnigan et al. computationally predicted a single evolutionary precursor for the two S. *cerevisiae* a-subunit isoforms (Anc.a) and then expressed this sequence in yeast (Finnigan et al., 2011). The Anc. a appears to support V-ATPase function in the Golgi and vacuole, based on complementation of growth phenotypes, and the authors speculated that slow transport through the Golgi and endosome en route to the vacuole allow it to provide sufficient acidification for both compartments. Lupanga et al. proposed a similar model in *Marchantia*, and demonstrated that the Golgi a1 isoform found in A. *thaliana* (which has three isoforms) was only essential for a limited period of development (Lupanga et al., 2020). The high level of a-subunit isoform diversity in many organisms suggests there might be a cost in versatility or regulatory capacity to having only one a-isoform, but this has not been addressed in detail. It may be that other V-ATPase subunit isoforms allow versatility of function in some organisms with a single a-subunit isoform, or it may be that some a-subunit isoforms are only essential under a limited number of conditions, as shown in *Arabidopsis* (Lupanga et al., 2020).

### 2.1 Yeast (S. *cerevisiae*) Vph1 and Stv1 as a paradigm for a-subunit isoform function

In S. *cerevisiae*, the only V-ATPase subunit with more than one isoform is the a-subunit, which is encoded by two genes, *VPH1* and *STV1* (Table 1). Deletion of any of the other V-ATPase subunit genes results in complete loss of V-ATPase activity and a conditional lethality known as the Vma^−^ phenotype (Kane, 2007). Specifically, yeast cells lacking V-ATPase activity can grow at an extracellular pH of 5, but fail to grow at pH 7.5 or in the presence of Ca^2+^ or multiple metals. In contrast, deletion of *VPH1* (*vph1∆* mutant) results in a partial Vma^−^ phenotype under most conditions, although *vph1∆* mutants are completely unable to grow in the presence of 4 mM Zn^2+^ because of the importance of vacuolar acidification for zinc detoxification. A *vph1∆stv1∆* double mutant exhibits a full Vma^−^ phenotype. Manolson et al. cloned both the *VPH1* and *STV1* genes and showed that Vph1 localized to the vacuole at steady state, while Stv1 localized to puncta later shown to be primarily Golgi (Manolson et al., 1992; Manolson et al., 1994). Because Vph1 transits to the vacuole through the Golgi, it can provide some Golgi acidification during its passage, and as a result, a *stv1∆* mutant has very little growth defect. Overexpression of *STV1* causes a portion of the Stv1 protein to mislocalize to the vacuole, where it can partially compensate for loss of *VPH1* (Manolson et al., 1994). However, several studies indicate that Stv1 and Vph1 have distinct catalytic and regulatory properties that can vary with their membrane environment (Kawasaki-Nishi, 2001b; Qi and Forgac, 2007).

**TABLE 1:** Distribution of human a-subunit isoforms. The isoform nomenclature, tissues where each isoform is expressed (along with tissues where expression may be enriched), and the cellular localization are listed for the human and yeast a-subunit isoforms.

	a-subunit isoforms	Expression	Localization	References
Human	a1 (ATP6V0a1)	Ubiquitous (enriched in brain)	Lysosomes, endosomes, and synaptic vesicles	Morel et al. (2003), Hurtado-Lorenzo et al. (2006), Bodzeta et al. (2017)
	a2 (ATP6V0a2)	Ubiquitous	Golgi and early endosomes	Hurtado-Lorenzo et al., 2006; Kornak et al., 2008; Zoncu et al., 2011)
	a3 (ATP6V0a3)	Ubiquitous (enriched in osteoclasts)	Secretory lysosomes	Toyomura et al. (2003), Sun-Wada et al. (2009), Matsumoto et al. (2018)
	a4 (ATP6V0a4)	Tissue-specific- Kidney, epididymis, and inner ear	Plasma membrane and vesicles	Oka et al. (2001), Pietrement et al. (2006)
Yeast	Vph1	Constitutive	Vacuole	Manolson et al. (1992)
	Stv1	Constitutive	Golgi and endosomes	Manolson et al. (1994)

Chimeras swapping the NT and CT domains between Vph1 and Stv1 provided important insights into where functional and localization information resides in the two isoforms (Kawasaki-Nishi, 2001a). These studies demonstrated that the NT domains of Vph1 and Stv1 are responsible for targeting V-ATPases the vacuole and Golgi, respectively. These experiments and others also suggested differences in catalytic properties between Stv1-and Vph1-containing V-ATPases; specifically, chimeras containing the Vph1CT domain were reported to show better coupling of ATP hydrolysis and proton transport (Kawasaki-Nishi et al., 2001; Kawasaki-Nishi et al., 2001).

As described in more detail below, Vph1-and Stv1-containing V-ATPases also have distinct regulatory properties, including binding to different phosphoinositide lipids, susceptibility to reversible disassembly, and dependence on the RAVE complex. Similarly, other a-subunit isoforms have distinct regulatory properties.

### 2.2 Human a-subunit isoforms

Human and other mammalian genomes generally contain four different a-subunit isoform genes. Three of the isoforms are ubiquitously expressed and one (a4) shows highly specific tissue expression (Table 1). The four major a-subunit isoforms are designated a1-a4 (encoded by the ATP6V0a1-ATP6V0a4 genes) in humans. a1 and a2 reside in intracellular membranes, with a1 occupying lysosomes and endosomes and a2 present in the Golgi and early endosomes. a1 is also enriched in brain, where it drives neurotransmitter uptake into synaptic vesicles and is found at the presynaptic plasma membranes of nerve terminals (Morel et al., 2003; Bodzeta et al., 2017). a2 appears to be a Golgi isoform but it localizes to endosomes and can acidify both locations (Hurtado-Lorenzo et al., 2006; Kornak et al., 2008; Zoncu et al., 2011). Expression of a1 appears to increase in cells with reduced levels of a2 and a3, suggesting there may be some compensation (McGuire et al., 2019). Partial cross-complementation among these isoforms has been observed previously (Matsumoto et al., 2018). a3 is distributed to lysosomes, and particularly secretory lysosomes that are capable of fusion with phagosomes or the plasma membrane of osteoclasts (Toyomura et al., 2003; Sun-Wada et al., 2009; Matsumoto et al., 2018). In undifferentiated osteoclast progenitor cells, a3 localizes to late endosomes and lysosomes, but these secretory lysosomes are trafficked to the plasma membrane during osteoclast maturation (Toyomura et al., 2003). Similarly, a3 appears to be responsible for both acidification of cytotoxic granules in cytolytic T Cells and transport of these granules to the immune synapse (Chitirala et al., 2020). Although a1, a2, and a3 are expressed in a broad range of tissues, a4 expression is restricted to the kidney, epididymis, and inner ear (Oka et al., 2001; Pietrement et al., 2006). V-ATPases containing a4 are localized to the apical plasma membrane of renal intercalated cells and epididymal clear cells where they function in urinary acidification and sperm maturation, respectively (Oka, Murata, et al., 2001; Pietrement et al., 2006). V-ATPases containing a4 are also found in the inner ear (Stover et al., 2002). Remarkably, all four a-subunit isoforms are expressed at distinct localizations very early (pre-gastrulation) in mouse embryos, suggesting that all four play a role in early development (Sun-Wada and Wada, 2022).

### 2.3 Human diseases associated with specific a-subunit isoforms

Complete loss of V-ATPase activity is lethal very early in development of mammals (Sun-Wada et al., 2003). However, mutations in each of the human isoforms have been described, with distinct clinical phenotypes. These phenotypes provide insights into the essential roles of each isoform, as well as the extent of redundancy and compensation between isoforms.

Mice containing a homozygous knockout of ATP6V0a1 die very early in development, similar to mice lacking V-ATPase subunits encoded by a single subunit isoform (Aoto et al., 2021) (Sun-Wada et al., 2003). This indicates that a1 plays an essential role early in mammalian development. However, humans heterozygous for *de novo* variants expected to have a complete loss of function (R741Q) or homozygous for mutant alleles expected to partially compromise function have been identified (Aoto et al., 2021). Mutations that seriously compromise the function of the a1 isoform result in multiple neurological phenotypes, including seizures, severe developmental delay, and encephalopathy (Bott et al., 2021). Introduction of these mutations into model systems and cell lines revealed multiple features that could contribute to the clinical phenotypes, including loss of lysosomal acidification, compromised autophagy, defects in synapse formation, and low levels of neurotransmitters in synaptic vesicles (Aoto et al., 2021) (Bott et al., 2021).

Loss of function mutations in ATP6V0a2 are associated with autosomal recessive cutis laxa (ARCL) and Wrinkly Skin Syndrome in humans. These relatively rare syndromes are characterized by loose, inelastic skin, accompanied by a number of developmental defects. Loss of V-ATPase function at the Golgi appears to account for many of these phenotypes. Compromised Golgi acidification arising from ATP6V0a2 mutations resulted in defective glycosylation and a number of intracellular trafficking defects (Hucthagowder et al., 2009; Fischer et al., 2012).

Loss of function mutations in ATP6V0a3 are associated with autosomal recessive osteopetrosis, which is characterized by brittle, overgrown bones and is generally fatal early in life. Most of the responsible mutations either prevent expression or destabilize the a3 protein (Chu et al., 2021).

Genetic loss of V-ATPase activity in distal renal tubule acidosis was first associated with mutations in an isoform of the V_1_ B-subunit (Karet et al., 1999), but was later shown to also occur with mutations in ATP6V0a4 (Stover et al., 2002). Mutations in either V-ATPase subunit can also cause sensorineural deafness, although deafness is often exhibited later in life than the acidosis.

## 3 Regulatory mechanisms involving a-subunit isoforms

Subunit isoforms offer a regulatory plasticity to V-ATPase function. This plasticity contributes to the versatility of V-ATPase functions and allows subpopulations of V-ATPases to respond to different stresses. A number of regulatory mechanisms with direct relationship to the a-subunit and its isoforms are addressed below.

### 3.1 Reversible disassembly

One of the unique features of eukaryotic V-ATPase is regulation by reversible disassembly. In this process, the V_1_ and V_o_ domains of the V-ATPase dissociate reversibly in response to stimuli. Dissociation prevents both ATP hydrolysis and proton pumping, effectively “turning off” the V-ATPases (Graf et al., 1996; Kane and Parra, 2000; Parra et al., 2000; Couoh-Cardel et al., 2015). Reversible dissociation was first discovered in the tobacco hornworm M. *sexta* and in yeast (Kane, 1995; Sumner et al., 1995). In yeast, V_1_ and V_o_ dissociate in response to glucose withdrawal, while in M. *sexta* disassembly occurs with nutrient depletion at a specific developmental stage. Disassembly and inactivation of V-ATPase activity in these cases is believed to preserve ATP under conditions of nutritional stress. In both cases, V_1_ and V_o_ remain intact as two separate sub-complexes, except for the removal of subunit C from both sectors (Kane, 1995; Tabke et al., 2014)). In yeast, after adding back glucose to glucose starved cells, the RAVE complex (Regulator of H^+^-ATPase of Vacuolar and Endosomal membranes) catalyzes the reassembly of both V_1_ and C with V_o_ (Seol et al., 2001; Smardon et al., 2002; Smardon and Kane, 2007). Significantly, reversible disassembly exhibits a-isoform specificity in yeast (Smardon et al., 2014). Vph1-containing V-ATPase complexes disassemble more readily upon glucose deprivation than Stv1-containing V-ATPases. However, some differences in disassembly were dependent on membrane environment. Kawasaki-Nishi et al. (Kawasaki-Nishi, 2001a) found that chimeras containing the Vph1NT and Stv1CT showed more disassembly upon glucose deprivation than those containing the Stv1NT and Vph1CT, implicating the Vph1NT domain in the process. Subsequently, the RAVE complex was shown to bind to Vph1NT but not Stv1NT (Smardon et al., 2014; Smardon et al., 2015). Loss of RAVE function results in almost complete loss of V-ATPase activity in isolated vacuoles, but overexpression of *STV1* allows recovery of activity, further indicating that V-ATPases containing Stv1 can assemble in the absence of RAVE (Smardon et al., 2014). Regions of RAVE capable of binding to Vph1NT, V_1_ subunit C, and V_1_ have been defined biochemically (Smardon et al., 2015). Later work demonstrated that the interaction of RAVE with Vph1NT was the central event in glucose-induced V-ATPase reassembly, as glucose addition to deprived cells could induce recruitment of RAVE to Vph1 at the vacuolar membrane even in the absence of V_1_ or subunit C (Jaskolka and Kane, 2020). Significantly, Rabconnectin-3 complexes present in higher eukaryotes share structural and functional homology with yeast RAVE (Jaskolka et al., 2021).

**FIGURE 2:**
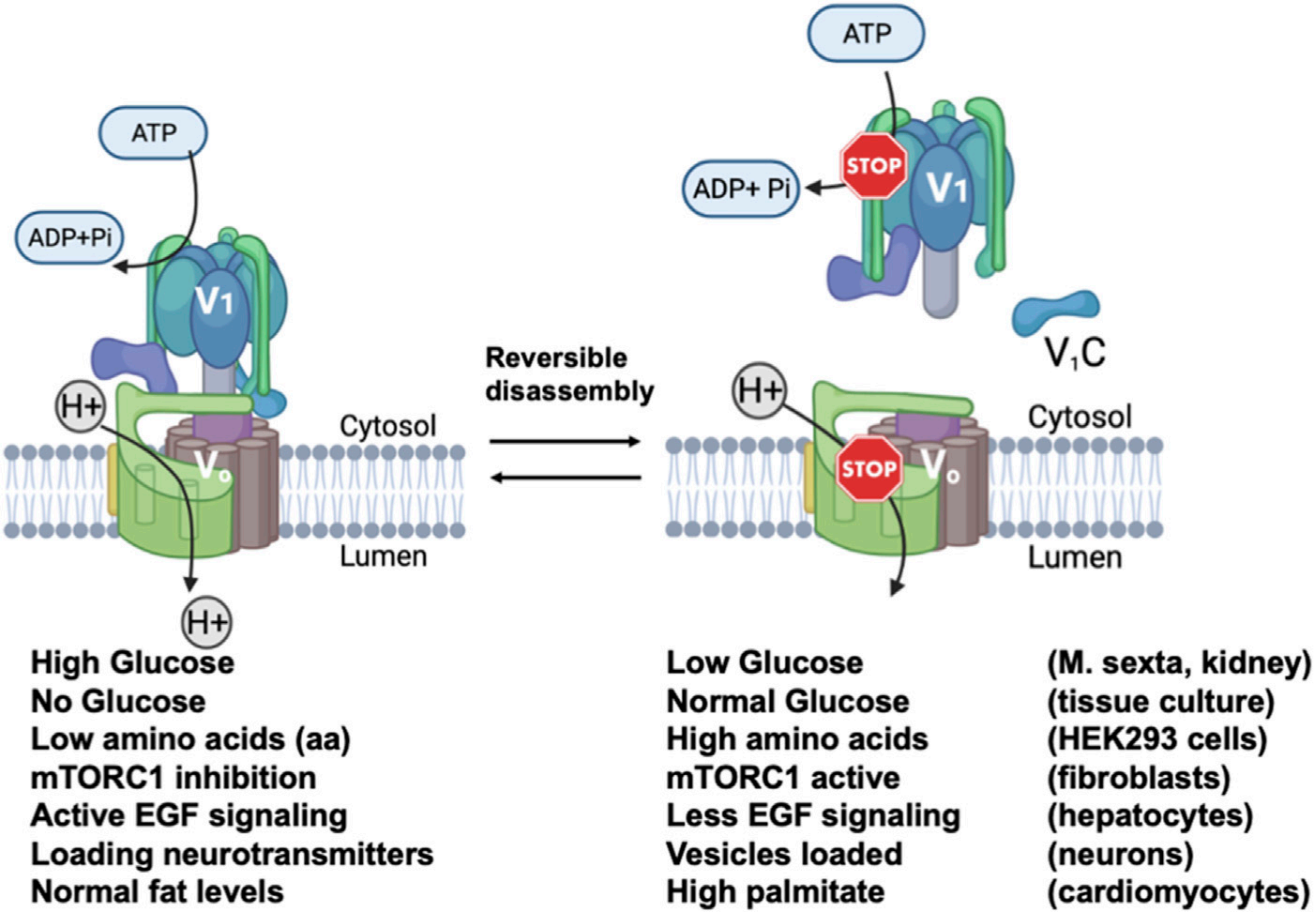
Reversible disassembly of V-ATPases in mammalian cells. Diagram showing the assembled and active V-ATPase (left) and the disassembled and inactive V-ATPase (right) are interconverted by reversible disassembly. Below the figure, conditions shown to cause reversible disassembly in mammalian cells are listed, with the conditions favoring the assembly listed beneath the assembled structure and the conditions favoring disassembly listed beneath the disassembled structure. The cell types in which each type of reversible disassembly was observed are listed at the right. References for each reversible disassembly condition are cited in the text. The V-ATPase diagrams were created using Biorender. This figure is adapted from Jaskolka et al., 2021.

Reversible disassembly occurs in mammalian cells in response to a diverse set of stimuli (Figure 2). Early studies in kidney showed increased assembly of cell surface V-ATPases in response to high extracellular glucose (Sautin et al., 2005), but in other cells, lysosomal V-ATPases were shown to assemble at very low glucose concentrations (McGuire and Forgac, 2018). In general, V-ATPase assembly is often stimulated in mammalian cells in order to augment the hydrolytic activity of the lysosome. In this context, dendritic cells increase V-ATPase assembly as they mature in order to decrease lysosomal pH, increase proteolysis, and favor antigen presentation ((Trombetta et al., 2003). Amino acid deprivation induces increased assembly of the V-ATPase and lysosomal acidification in order to support proteolysis and mobilize amino acids from the lysosome (Stransky and Forgac, 2015). More recently, fibroblasts undergoing starvation-induced macropinocytosis were shown to increase V-ATPase assembly and decrease lysosomal pH upon mTOR inhibition (Ratto et al., 2022). In this setting, lysosomal protease activity was increased with V-ATPase assembly. Increased V-ATPase activity supports the recycling capability of lysosomes since degradation of macromolecules releases amino acids and other nutrients. Epidermal growth factor (EGF) stimulates V-ATPase assembly in endosomes and lysosomes; in this setting, increased V-ATPase activity activates mTOR and may help generate nutrients by lysosomal hydrolysis in anticipation of increased cell growth (Xu et al., 2012). Ratto et al. (Ratto et al., 2022) demonstrated involvement of the a3 isoform and the mammalian Rabconnectin-3 complex in V-ATPase reassembly during macropinocytosis. However, in most cases, the specific a-isoforms involved in reversible disassembly at the late endosomes and lysosomes have not been defined. Given the enrichment of the a1 and a3 isoforms in the late endosome and lysosome, it is likely that both could be involved in different settings.

Reversible disassembly is associated with less adaptive responses to environmental challenges as well. In cardiomyocytes, V-ATPases in the early endosome disassemble in response to lipid overload, resulting in endosome alkalinization, relocalization of the fatty acid transporter CD36 to the plasma membrane, and further uptake of extracellular lipids (Liu et al., 2017). This process is highly significant, as it appears to be an early step in insulin resistance and ultimately cardiac contractile dysfunction (Liu et al., 2017) (Wang et al., 2022). In cultured cells, promoting V-ATPase reassembly restored endosomal CD36 localization and reduced lipid uptake (Wang et al., 2021; S. Wang et al., 2020). In cardiomyocytes, the V-ATPases involved in reversible disassembly contain the a2 isoform (Liu et al., 2017). Therapeutic manipulation of V-ATPase assembly has been proposed for influenza A infection as well (Kohio and Adamson, 2013). Acidification of the endosome is critical for release of the viral genome, and at low glucose levels, where V-ATPases disassemble, viral progression is compromised (Kohio and Adamson, 2013).

In neurons, reversible disassembly may play a critical and constitutive role in synaptic vesicle loading, fusion, and recycling. V-ATPase activity is critical for loading neurotransmitters into synaptic vesicles. The synaptic vesicle V-ATPase contains the a1 isoform (Poea-Guyon et al., 2013). Bodzeta et al. (Bodzeta et al., 2017) showed that V-ATPases are assembled to drive neurotransmitter uptake, followed by disassembly of the V_1_ from the loaded vesicle in preparation for fusion (Morel, 2003). Upon endocytosis from the plasma membrane and reformation of synaptic vesicles, V-ATPases reassembled to support the next round of the synaptic vesicle cycle. V-ATPase assembly appears to respond to the luminal pH of the vesicle in this context (Bodzeta et al., 2017).

While it is clear that reversible disassembly is a central regulatory mechanism for V-ATPases in higher eukaryotes, the signaling pathways involved are complex and only partially elucidated (reviewed in (Collins and Forgac, 2020)). In addition, the specific a-subunit isoforms involved have only been defined in a few cases. Although Rabconnectin-3 complexes have been implicated in reversible disassembly in mammalian cells, it is not known whether these complexes exhibit isoform specificity, as shown for the RAVE complex and yeast V-ATPase. In addition, there are multiple subunit isoforms in mammalian Rabconnectin-3 complexes (Jaskolka et al., 2021), suggesting rich potential for a-isoform specificity.

### 3.2 Binding to glycolytic enzymes

The relationship between the V-ATPase and glycolysis is complex and bidirectional (Hayek et al., 2019). Direct interactions between V-ATPases and glycolytic enzymes have been observed in multiple organisms (Hayek et al., 2019), and many of these interactions involve the a-subunit. These interactions have been suggested to provide a direct supply of ATP from a glycolytic metabolon to the V-ATPase (Lu et al., 2001). However, as described below, there is still much to be learned about how these interactions actually affect the V-ATPase.

Phosphofructokinase (PFK) catalyzes the third step of glycolysis, where it utilizes ATP to drive the conversion of fructose-6-phosphate to fructose-1,6-bisphosphate. In human renal cells, PFK-1 binds in last 45 amino acids of a4 (Su et al., 2003). Significantly, two missense mutations in this region are associated with recessive distal renal tubule acidosis, suggesting that they may interfere with V-ATPase activity (Smith et al., 2000; Stover et al., 2002). These two disease-associated mutations are in highly conserved amino acids, and the mutations were introduced at the corresponding location in yeast Vph1. The mutations disrupted V-ATPase activity, supporting the functional importance of the interaction between PFK-1 interaction and the a4 isoform (Su et al., 2008). PFK-1 also binds to the V-ATPase in yeast (Chan and Parra, 2014). Both the Pfk1 and Pfk2 subunits of PFK-1 are able to bind, but the site of interaction on the V-ATPase was not determined (Chan and Parra, 2014). Deletion of the Pfk2 subunit of yeast PFK-1 reduces downstream glycolytic intermediates significantly more than Pfk1p (Heinisch, 1986). *pfk2∆* mutants have defects in vacuolar acidification (Chan and Parra, 2014) and exhibit a partial Vma^−^ phenotype; *pfk1∆* mutants have milder effects. The *pfk2∆* mutant showed reduced reassembly of the V-ATPase after glucose readdition, suggesting a potential role for Pfk2 in signaling carbon source availability to the V-ATPase. Interestingly, increasing glycolytic flux by adding higher levels of glucose reversed this reassembly defect (Chan and Parra, 2014).

The glycolytic enzyme aldolase has been reported to bind to V-ATPases in yeast (Lu et al., 2007), plants (Konishi et al., 2004), and mammals (Merkulova et al., 2011). Lu et al. reported that aldolase binds directly to the yeast V_1_ subunits B and E and the a4NT domain *in vitro* (Lu et al., 2004). Despite the presence of potential binding sites in both the V_1_ and V_o_ sectors, aldolase bound only to the intact V-ATPase in the presence of glucose, and failed to bind to the separate V_1_ and V_o_ sectors in the absence of glucose (Lu et al., 2004). Konishi et al. reported co-immunoprecipitation of aldolase from rice roots with antibodies to the V-ATPase (Konishi et al., 2004). Merkulova et al. reported an indirect interaction between V-ATPases containing multiple a-subunit isoforms and aldolase via the Arf6 GTP exchange factor, ARNO (Merkulova et al., 2011). The functional effects of aldolase interaction with the V-ATPase are still not clear. In yeast, V-ATPases are disassembled and inactive in aldolase deletion mutants (Lu et al., 2001). However, interpretation is complicated by the severe glycolytic defects of the aldolase mutant, since failure to metabolize glucose would mimic glucose deprivation and thus drive disassembly. Subsequent studies comparing effects of overexpressing an inactive aldolase mutant and an active aldolase mutant defective for binding to the V_1_B subunit suggested that binding, but not aldolase activity, was important for V-ATPase activity (Lu et al., 2007). At present, it is clear that aldolase binds to V-ATPases across organisms, but further work is necessary to define its functional role.

### 3.3 Lipid interactions

**FIGURE 3:**
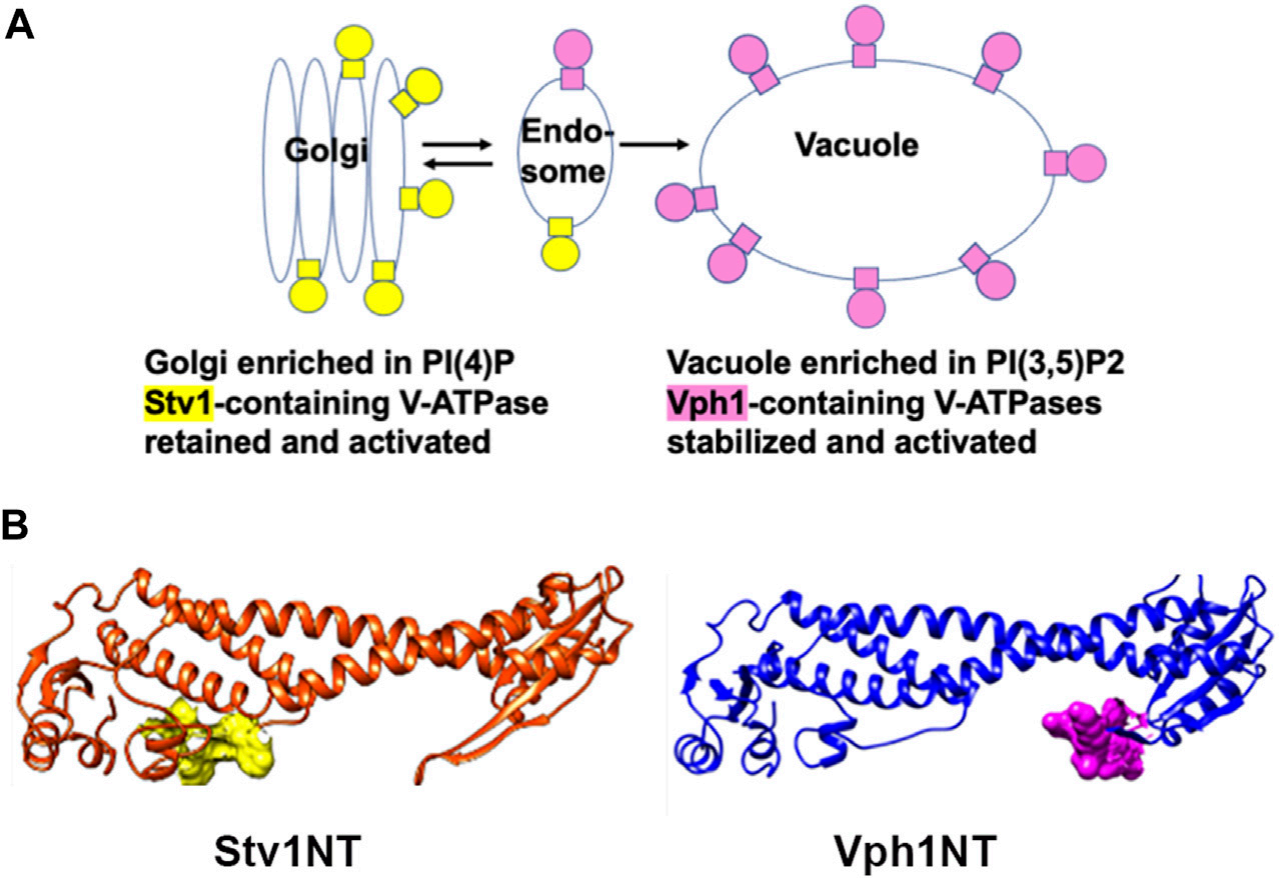
Organelle-specific PIP lipids bind to yeast a-subunit isoforms. **(A)** Diagram showing organelle-specific distribution of PIP lipids PI(4)P and PI(3,5)P2 and V-ATPases containing distinct a-subunit isoforms. **(B)** Stv1NT and Vph1NT structures showing binding sites for PI(4)P in the proximal end of Stv1NT (yellow) and PI(3,5)P2 in the distal end of Vph1NT (pink).

The V_o_ sector of the V-ATPase is embedded in the lipid membrane, but recent data indicate that interactions of the a-subunit with specific lipid headgroups can have regulatory effects (Banerjee and Kane, 2020). Specific phosphoinositide phospholipids (PIPs) are enriched in certain organelles and membrane domains (Figure 3A). The Golgi apparatus is generally enriched in PI4P. Reduction of PI4P levels *in vivo* causes Stv1-containing V-ATPases to relocalize to the vacuole, implicating PI4P in Golgi retention or recycling of Stv1 (Banerjee and Kane, 2017). The N-terminal domain of Stv1 (Stv1NT) was shown to bind tightly to PI4P-containing liposomes *in vitro*, and mutation of a sequence in the proximal end of Stv1NT (W83KY) also abrogated PI4P binding *in vitro* (Banerjee and Kane, 2017) (Figure 3B). Significantly, mutations in this sequence were previously shown to alter Stv1 localization (Finnigan et al., 2012). These data indicate that interaction of Stv1 with PI4P helps to localize Stv1-containing V-ATPases to the Golgi. Later work suggested that ATPase activity of solubilized V-ATPase complexes containing Stv1 could also be activated by addition of PI4P lipids (Vasanthakumar et al., 2019).

PI(3,5)P_2_ is a low level signaling lipid found primarily in late endosomes and vacuoles (lysosomes in mammalian cells) where Vph1-containing V-ATPases are localized (Gary et al., 1998; Jin et al., 2016). In yeast mutants defective in PI(3,5)P_2_ synthesis, V-ATPases containing the Vph1 isoform still localize to the vacuole, but have reduced activity and assembly (Li et al., 2014b). PI(3,5)P_2_ levels are transiently increased by up to 20-fold in response to osmotic stress (Dove et al., 1997; Duex et al., 2006); V-ATPase activity is activated in a PI(3,5)P_2_ dependent manner under these conditions (Li et al., 2014b). Unlike Stv1NT, Vph1NT did not show significant binding to liposomes containing PI(3,5)P_2_ *in vitro* (Tuli and Kane, 2023). However, addition of short chain lipids with a PI(3,5)P_2_ headgroup to isolated vacuolar vesicles activates V-ATPase activity and proton pumping (Banerjee et al., 2019). Using this assay system, it was possible to identify mutations in Vph1NT that compromise activation. Two sequences in a loop of distal domain of Vph1NT prevented PI(3,5)P_2_-dependent activation *in vitro* (Figure 3B), as well as tolerance of osmotic stress *in vivo* (Banerjee et al., 2019).

These data in yeast suggest that PIP lipids can provide organelle-specific regulation of V-ATPase. Although there is substantial evidence linking PIPs to organelle acidification (Banerjee and Kane, 2020), it is still not clear whether a-isoform-specific binding to PIPs is conserved in higher eukaryotes. Finally, it is also notable that PI(3,5)P_2_ plays a critical role in vacuolar acidification in *Arabidopsis*, but acts on a chloride channel responsible for balancing electrogenic proton transport by the V-ATPase, rather than the V-ATPase itself (Carpaneto et al., 2017). In contrast, PI(3,5)P_2_ actually inhibits a chloride channel in mammalian lysosomes, and loss of this inhibition results in hyperacidification (Leray et al., 2022). Clearly, PIPs can exert both direct and indirect effects on V-ATPase activity and organelle acidification.

An entirely different type of lipid regulation of the a1 isoform was observed in mouse models of the neurodegenerative disease INCL (infantile neuronal ceroid lipofuscinosis). This disease arises from loss of function of a palmitoyl transferase, CLN1, and mice lacking CLN1 exhibited poor trafficking of the a1 isoform to lysosomes and defective lysosomal acidification. Curiously, it was found that CLN1 catalyzed covalent palmitoylation of cysteine 25 of the a1 isoform. Loss of this modification perturbed the transport of a1-containing V-ATPases to the lysosome, apparently by preventing interactions with clathrin adaptor proteins (Bagh et al., 2017). One puzzling aspect of this modification is that current structures of mammalian V-ATPases containing the a1 isoform (Abbas et al., 2020; L. Wang et al., 2020; R. Wang et al., 2020; S. Wang et al., 2020) place cysteine 25 at some distance from the membrane. Further work could address how palmitoylation affects V-ATPase structure and function, as well as trafficking.

### 3.4 Interactions with trafficking components

As described above, different a-subunit isoforms play a major role in directing V-ATPase complexes to their cellular destinations, so it is not surprising that there is interplay between cellular trafficking machinery and the V-ATPase. However, these interactions are not simple and are still not completely understood in most cases.

V-ATPase interaction with the cytoskeleton was first reported in 1997 (Nakamura et al., 1997). Osteosclerotic mice harbor a mutation in the a3-subunit (Scimeca et al., 2000), and V-ATPases in these mice did not interact with cytoskeleton. As a result, the cells failed to recruit V-ATPases to the ruffled border membrane. This finding suggested a functional interaction between V-ATPases and the cytoskeleton. A direct interaction between V-ATPase and microfilaments was detected in osteoclasts and reconstituted using isolated kidney V-ATPases (Lee et al., 1999). Later studies showed B-subunit of V-ATPases also contains a high affinity microfilament binding site (Holliday et al., 2000), suggesting that multiple subunits may support interactions with the cytoskeleton. In cytolytic T Cells, knockdown of the a3 isoform reduced interaction of cytotoxic granules with the microtubule network, preventing their transport (Chitirala et al., 2020). In contrast to examples of direct interaction with the actin or microtubule cytoskeleton driving V-ATPase localization, recruitment of V-ATPases containing the Vo a4 isoform to the plasma membrane of epididymal clear cells appears to require depolymerization of the actin cytoskeleton by gelsolin (Beaulieu et al., 2005). Significantly, in each of the examples described above, the cytoskeleton is involved in recruitment of V-ATPases to the plasma membrane. Less is known about cytoskeletal interactions with V-ATPases in organelles such as lysosomes and Golgi, but disassembly of V-ATPases in yeast has been reported to require microtubules (Xu and Forgac, 2001).

The small GTPase Arf6 and its cognate GDP/GTP exchange factor ARNO are involved in recognition of luminal pH in early endosomes and regulation of endocytic traffic (D'Souza-Schorey et al., 1995; Donaldson, 2003). Specifically, V-ATPase mediated endosomal acidification is essential for the recruitment of the small GTPase ARNO from cytosol to endosomal membranes (Maranda et al., 2001; Marshansky et al., 2002). It was proposed that luminal pH was sensed by the a2CT domain, generating conformational information that was passed through the a2NT domain to ARNO (Hurtado-Lorenzo et al., 2006). ARNO binds to a2NT, and Arf6 interacts with proteolipid c-subunits of the V-ATPase. Loss of V-ATPase activity disrupts both endosomal acidification and V-ATPase interaction with ARNO/Arf6 and ultimately, impairs endocytic trafficking (Hurtado-Lorenzo et al., 2006). A later study reported that the interaction between the a2-subunit of V-ATPase and ARNO is complex, involving various binding sites on both proteins (Merkulova et al., 2010). Homology modeling revealed two ARNO binding sites located on the distal and proximal lobes of a2NT. The Sec7-domain of ARNO has a major interaction site on the proximal lobe of a2NT, whereas the PH-domain of ARNO binds to the distal lobe of aNT. Structural studies suggest that the two ARNO binding sites are in a close proximity to two, EG binding sites on a-subunit but are not identical (Marshansky et al., 2019). These ARNO binding sites are likely to be accessible in the assembled enzyme and could help recruit ARNO to the intact V-ATPase complex. In support of this, the intact S. *cerevisiae* V-ATPase interacts with a truncated form of ARNO (Hosokawa et al., 2013). However, the binding of ARNO to a2 may compromise the stability of interaction between the a2-subunit and the, EG peripheral stalks. Thus, the acidification-dependent recruitment of ARNO to V-ATPase could be involved in regulating disassembly of intact V-ATPase complex as suggested by the structural models (Marshansky et al., 2019). This possibility has not been confirmed. In addition to the a2 isoform, a1-, a3-and a4-isoforms have also been shown to interact with ARNO *in vitro* (Merkulova et al., 2011), suggesting that ARNO interactions could affect V-ATPases in multiple cellular locales and may not be entirely isoform-specific.

The V_o_ domains of V-ATPases have also been implicated in membrane fusion, and in this context, a-subunits have been reported to interact with SNARE proteins. Initial studies (Peters et al., 2001) indicated that V_o_ complexes containing Vph1, separate from V_1_, could facilitate fusion between vacuoles. This initial work suggested that V_o_-mediated fusion occurred after SNARE pairing. However, subsequent work in flies implicated a direct interaction between SNARE proteins and the V_o_ a-subunit in synaptic vesicle exocytosis (Hiesinger et al., 2005; Williamson et al., 2010). In addition, the oc/oc mouse, which does not express the a3 isoform, is able to load insulin-containing secretory granules, but cannot secrete insulin from beta cells. This suggests an acidification-independent function that could be related to vesicle fusion (Sun-Wada et al., 2006). However, the role of V_o_ subcomplexes in fusion has been questioned. Experiments in yeast showed that acidification of vacuoles by another enzyme could support vacuole-vacuole fusion. This indicated that acidification by the V-ATPase, not the V_o_ structure itself, was important for fusion (Coonrod et al., 2013). A later study identified an acidification-independent role of V_o_ a1 which is regulated by Ca^2+^– calmodulin (Wang et al., 2014). In *Drosophila*, calmodulin binds tightly to the aNT domain of a1 isoform in a calcium-dependent manner; this interaction was found to be critical for the regulation of the a1 subunit in neurons (Zhang et al., 2008). Bodzeta et al. (Bodzeta et al., 2017) argued that the V-ATPases are essential for neurotransmitter loading, but not for synaptic vesicle fusion. Thus, there is substantial evidence that suggests that the V_o_ subcomplex may be involved in certain membrane fusion events, but it is clear that more work is required to confirm the mechanism and the relationship to proton transport.

### 3.5 Defining the role of aNT domains in isoform-specific stress responses

Because of the complexity of V-ATPase structure, regulatory mechanisms have often been defined and investigated individually. However, it is important to recognize that V-ATPase a-subunit isoforms, and particularly the aNT domains, have the capacity to respond to multiple different regulatory inputs. To narrow down the regions of yeast Stv1NT and Vph1NT involved in different modes of regulation, chimeras of Vph1NT and Stv1NT were constructed by swapping the proximal and distal domains between the two isoforms (Tuli and Kane, 2023). Biochemical characterization of these two aNT chimeras *in vitro* indicated that RAVE binding is likely encoded in the proximal end of Vph1NT. However, binding to phosphoinositide lipids proved to be complex. Specifically, the SPVD (Stv1 proximal Vph1NT distal) chimera bound more tightly to both PI4P and PI(3,5)P_2_ than either Stv1NT or Vph1NT, suggesting that multiple aNT sequences can be involved in phosphoinositide binding. When the chimeric aNTs were expressed in yeast as part of full-length a-subunits containing Vph1CT, the SPVD chimera supported wild-type activity in isolated vacuoles, even though this chimera lacks RAVE binding (Tuli and Kane, 2023). In yeast, V-ATPases are regulated both in response to shifts in carbon source, by reversible disassembly, and in response to high extracellular pH, which requires PI(3,5)P_2_ binding (Kane, 1995; Li et al., 2014a). The SPVD chimera proved to respond slowly to shifts in carbon source, but after an initial lag, grew more rapidly than wild-type cells after a shift to high extracellular pH (Tuli and Kane, 2023). These results highlight the presence of multiple regulatory inputs targeting a-subunit isoforms and the physiological importance of integrating these inputs in response to different stress conditions.

**FIGURE 4:**
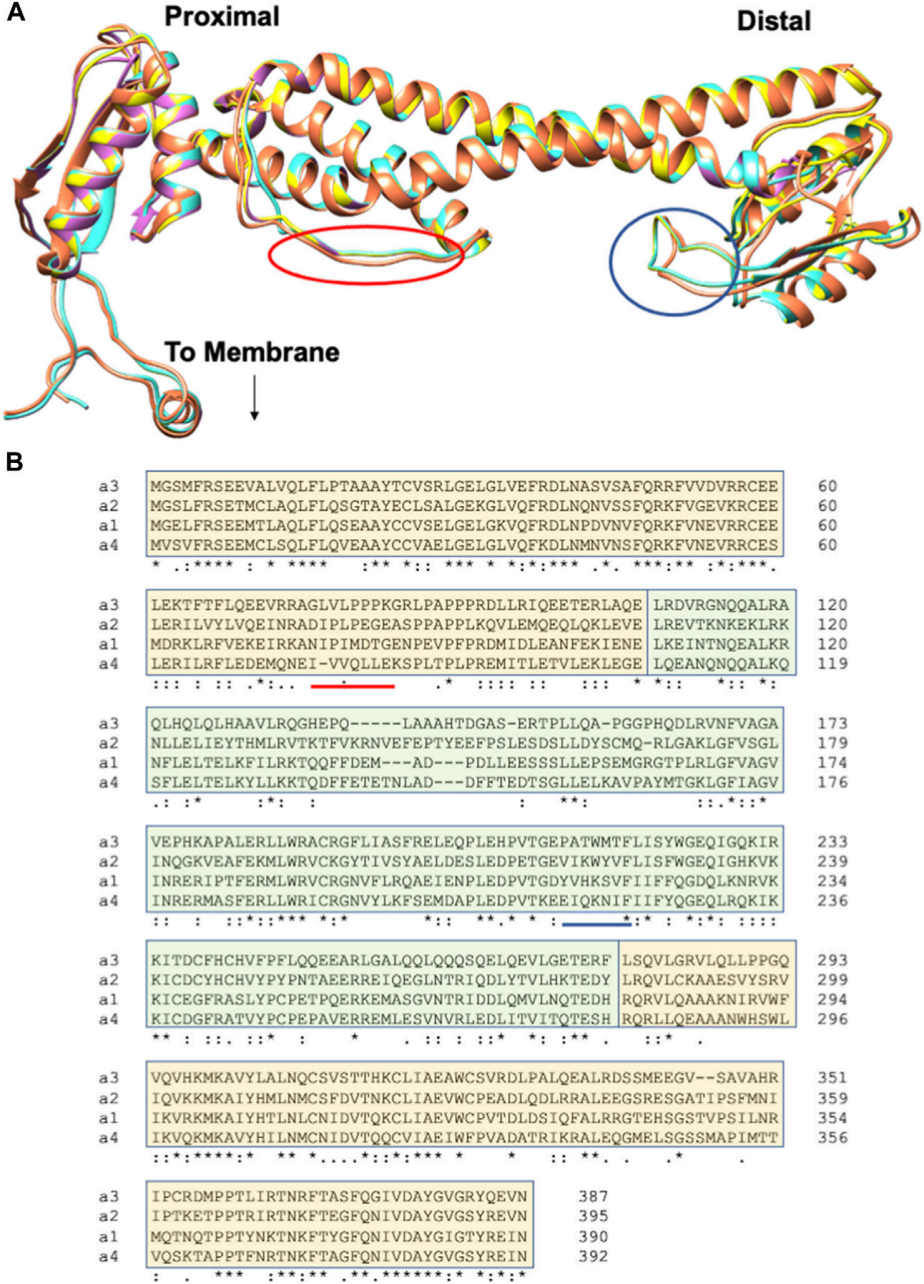
Human aNT isoforms exhibit similar structures but variation in sequences. **(A)** An overlay of the cryo-EM structure of the a1NT (coral) and Phyre2 generated homology models of the a2NT, a3NT, and a4NT (yellow, orchid and turquoise, respectively). a2NT, a3NT, and a4NT are modeled on the mammalian rat brain V-ATPase (Abbas et al., 2020). The proximal end, distal end, and the point at which aCT binds (to membrane) are indicated. Colored circles indicate the membrane-oriented loops that would correspond to the loops containing PIP binding sites in yeast. **(B)** Multiple sequence alignment of the human aNT isoforms. Sequences contributing to the proximal end are highlighted in light orange; the distal end sequences are in light green. Regions of the sequence that would correspond the circled loops in **(A)** are underlined.

The *in vitro* approaches used to identify regulatory features in the two yeast aNT isoforms could help also define how human aNT isoforms drive distinct modes of regulation. aNT isoforms of all types are constrained by mechanistic demands of V-ATPase function, including binding to two distinct peripheral stalks; this likely accounts for the high degree of predicted structural similarity in Figure 4A. However, the aNT isoforms also encode critical isoform-specific regulatory information. The multisequence alignment of the four human aNT isoforms (Figure 4B) reveals regions of significant sequence divergence that can be mapped onto the conserved backbone structure and ultimately linked to different regulatory mechanisms. For example, two areas of sequence variability are indicated in the alignment and the structure that correspond to the regions where PIP lipids bind to Stv1 (proximal) and Vph1 (distal) shown in Figure 3B. The four aNT isoforms could be expressed in *E. coli* and tested for PIP-specific liposome binding, as reported for Stv1 and Vph1. If differences in PIP binding are observed for the aNT isoforms *in vitro*, these variable regions could be targeted for mutagenesis to determine the amino acids involved. Similarly, specificity for rabconnectin-3 subunit isoforms could be assessed using expressed proteins or other techniques like the yeast two-hybrid assay. As individual isoform-specific regulatory interactions emerge, they could be further dissected by construction of chimeric aNT domains. Through these experiments, the hierarchy of regulatory interactions can be mapped onto the structures of a-subunit isoforms and informative tools for *in vivo* analysis of isoform regulation can be made available.

## 4 Future perspectives for a-subunit isoforms

It has long been recognized that V-ATPase activity could be an important therapeutic target. Beyond the genetic diseases arising from loss of V-ATPase a-subunit isoforms described above, aberrant V-ATPase activity is very important in cancer (Stransky et al., 2016; Pamarthy et al., 2018). The a3 and a4 isoforms have been shown to relocalize to the plasma membrane of breast cancer cell lines from humans and mice, respectively (Capecci and Forgac, 2013; Cotter et al., 2016; McGuire et al., 2019). The essential role of a4 relocalization in tumor growth and metastasis was recently reinforced by studies of breast cancer xenografts in mouse model (Su et al., 2022). In ovarian cancer, the a2 isoform is particularly important (Kulshrestha et al., 2015). These and other results suggest that understanding why these a-subunit isoforms are overexpressed in cancer cells and how they direct V-ATPases to the plasma membrane could facilitate therapeutic targeting. In addition, future therapeutic approaches could also target the distinct regulatory features of V-ATPases containing these isoforms.

V-ATPase inhibitors are also very effective at blocking viral replication (Muller et al., 2011; Yeganeh et al., 2015). Although the toxicity of V-ATPase inhibitors has been viewed as a major impediment to therapy, it was possible to block viral replication in human lung epithelial cells with bafilomycin A1 at very low concentrations where lysosomal acidification remained intact (Yeganeh et al., 2015). Such studies suggest it may be possible to target V-ATPase subpopulations, even with existing drugs that are known to have broad specificity for V-ATPase isoforms, but there is no doubt this will be difficult. Alternatively, it may also be possible to target V-ATPase regulators that are particularly important for certain subpopulations. For example, a recent CRISPR screen demonstrated that knockout of the rabconnectin-3 subunit WDR7 was highly effective in preventing influenza entry (Li et al., 2020).

With the advent of V-ATPase structures and genomic studies that identify the full set of V-ATPase isoforms, as well as a growing collection of isoform-specific regulators, new opportunities will emerge for therapeutic targeting of specific V-ATPase subpopulations. Taking full advantage of these opportunities requires further research into the distribution of V-ATPase isoforms and their ability to functionally compensate for each other. Given their diversity and established role in regulation, a-subunit isoforms could directly provide a route to targeting V-ATPase sub-populations responsible for disease processes. However, if a-isoform localization varies significantly among different cell types, there may well be unexpected side effects. In addition, compensation among a-subunit isoforms has already been observed and could confound effective inhibition aimed at individual isoforms. Because there are isoforms of several V-ATPase subunits in mammalian cells, it may also be possible to target tissue- or cell-specific combinations of these isoforms. This will require more detailed analysis of V-ATPase isoform composition in distinct cell types and their localization within those cell types. However, these data should be accessible with current mass spectrometry techniques. Knowledge of isoform composition in different locales could be complemented by continued structural work to visualize V-ATPases with different isoform composition at high resolution or in complex with different regulators. Such studies will open additional opportunities for structure-based drug design. In summary, highly isoform- or cell-specific V-ATPase regulators could be therapeutic targets that would avoid the toxicity of complete V-ATPase inhibition. These directions represent the next Frontier in exploring the functional versatility and therapeutic potential of V-ATPase a-subunits.
